# Randomized cross-over trial of demand oxygen delivery system in nocturnal hypoxemia

**DOI:** 10.1097/MD.0000000000020031

**Published:** 2020-05-08

**Authors:** Tatsuya Nagano, Kazuyuki Kobayashi, Takashi Omori, Takehiro Otoshi, Kanoko Umezawa, Naoko Katsurada, Masatsugu Yamamoto, Motoko Tachihara, Yoshihiro Nishimura

**Affiliations:** a Division of Respiratory Medicine, Department of Internal Medicine, Kobe University Graduate School of Medicine; b Clinical and Translational Research Center, Kobe University Hospital, 7-5-2 Kusunoki-cho, Chuo-ku, Kobe, Hyogo, Japan.

**Keywords:** auto-demand, noninferiority, oxygen concentrator, randomized

## Abstract

**Background:**

It has not been determined that demand valve oxygen therapy is effective for nocturnal hypoxia. A portable oxygen concentrator with an auto-demand oxygen delivery system (auto-DODS; standard, high, and extra high) has recently been developed to improve oxygenation and comfort. This oxygen concentrator can supply a pulsed flow when it detects apnoea. The aim of this study is to demonstrate that this newly developed portable oxygen concentrator with an auto-demand function is non-inferior to a continuous-flow oxygen concentrator for nocturnal hypoxemia.

**Methods:**

Twenty patients with chronic obstructive pulmonary disease or interstitial pneumonia will be randomized to receive a portable oxygen concentrator with an auto-DODS or a continuous-flow oxygen concentrator during sleep. The primary endpoint is mean oxygen saturation (SpO_2_) during the total sleep time. The secondary endpoints are the ratios of time that the oxygen concentrator spends in each sensitivity mode (standard, high, and extra-high) and at a constant pulse rate to the total sleep time, the total time and ratio of time for which SpO_2_ is less than 90% during the total sleep time, the lowest value of SpO_2_ during the total sleep time, the mean and highest pulse rate during the total sleep time, the apnoea index during the total sleep time, the total sleep duration itself, and comfort and reliability as measured by numerical rating scale and questionnaires.

**Discussion:**

If the auto-DODS demonstrates non-inferiority to continuous flow in oxygenation during sleep, the auto-DODS can be used even at night, and the patient will need only 1 device.

**Trial Registration:**

The study was registered on Aug 23, 2019 (jRCTs052190042).

## 1 Introduction

Long-term oxygen therapy (LTOT) is reported to improve hypoxemia and survival and has become an essential treatment for patients with respiratory failure caused by chronic obstructive pulmonary disease (COPD), interstitial pneumonia, and other conditions.^[1]^ There are 3 types of LTOT: gaseous oxygen, portable liquid oxygen systems, and stationary oxygen concentrators. Portable liquid oxygen systems are generally used outside the home, and stationary oxygen concentrators are used at home. A respiration synchronizer (demand oxygen delivery system [DODS]) is a device that supplies oxygen from the oxygen cylinder only when the user is inhaling, thereby saving oxygen and extending the usable life of the oxygen cylinder by 2 to 3 times. Tiep et al compared DODS oxygen and continuous-flow oxygen in 10 chronic respiratory disease patients. At rest, the DODS was equivalent to continuous flow: oxygen saturation (SpO_2_) was 93.7% ± 2.1% with an DODS and 93.8% ± 1.9% with continuous flow. During exercise at corresponding settings, the SpO_2_ with low flow through a DODS was 90.5% ± 3.8%, the SpO_2_ with high flow through a DODS was 92.5% ± 2.8% and the SpO_2_ with continuous flow was 93.1% ± 3.1%.^[2]^ Garrod et al conducted a randomized controlled trial of a continuous-flow system and a DODS in 14 COPD patients and revealed that there was no significant difference in the mean arterial oxygen saturation between patients who used the DODS and those who received continuous-flow oxygen (*P* = .33). Furthermore, there was no significant difference between the distance walked using oxygen delivered at 2 L/min by continuous flow and via the DODS (*P* = .72; confidence interval [CI] 0.34–1.08).^[3]^ The conventional demand device is a portable oxygen concentrator and has a conventional demand mode. This conventional demand device detects the negative pressure gradient of inspiration and supplies oxygen only during inspiration, sounding an alarm only if there is no breathing for 3 minutes. On the other hand, the newly developed auto-demand device is an oxygen concentrator that has an auto-demand mode. This auto-DODS can automatically switch among 3 sensitivity modes – extra-high, high and standard – according to the negative pressure and the supply of oxygen specifically at the time of inhalation. Moreover, if there is no breathing for a fixed time, the system can deliver oxygen at a constant pulse rate in addition to sounding an alarm.

## 2 Material and methods

### 2.1 Aim

The aim of this study is to examine whether clinically important indices during sleep are comparable between oxygen delivery by an auto-DODS and by continuous flow.

### 2.2 Design and setting

**Figure 1:**
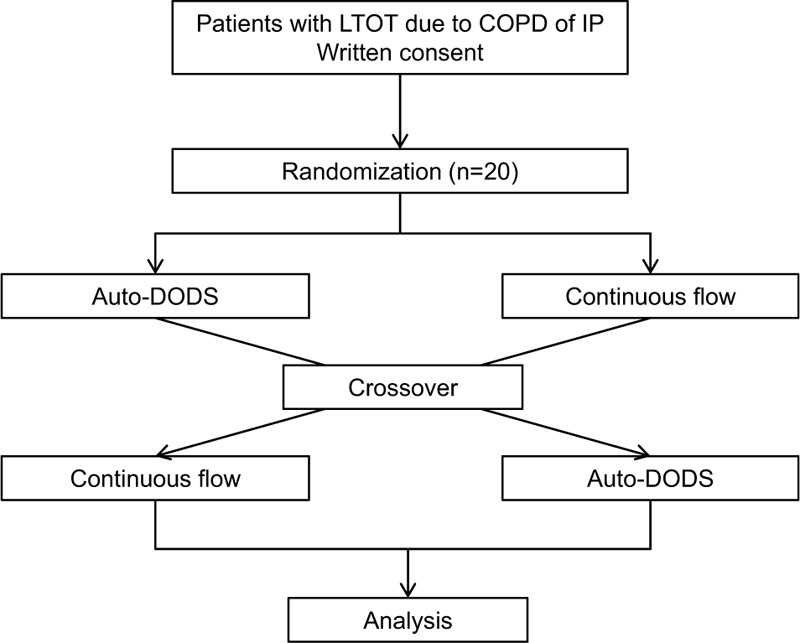
Flow chart of the study. COPD = chronic obstructive pulmonary disease, DODS = demand oxygen delivery system, IP = interstitial pneumonia, LTOT = long-term oxygen therapy.

All study endpoints are listed as bullet points in Table 1. This is a randomized open-label crossover noninferiority trial of an auto-DODS versus continuous flow during sleep by patients who are prescribed LTOT for COPD or interstitial pneumonia. In the present study, patients will use an auto-DODS or continuous flow at night in random order. Patients will be hospitalized for 2 nights and use different devices on the first and second nights. The auto-DODS will not be used during the day. Therefore, no carryover effect will occur. The data will be collected in the Kobe University Hospital in Kobe, Japan. The study design will be outlined in Figure 1.

**Table 1:**
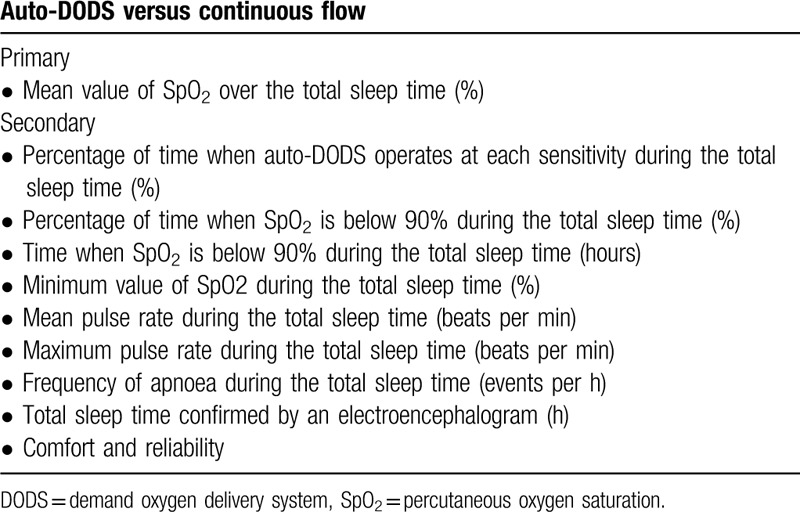
Primary and secondary endpoints addressed by this study protocol.

### 2.3 Participants

The inclusion and exclusion criteria for the trial are listed in Table 2. Participants who are outpatients of the Kobe University Hospital will be recruited.

**Table 2:**
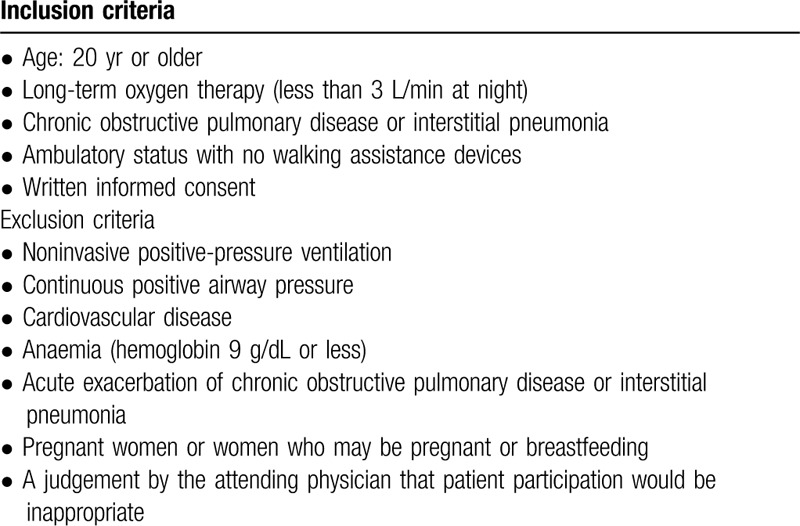
Inclusion and exclusion criteria.

### 2.4 Materials

Patients who are enrolled in this clinical trial and use the test devices will be analyzed. According to the results of the previous study using continuous flow and an auto-DODS (the sensitivity was fixed at extra-high) at night for LTOT patients, the difference in the mean value of SpO_2_ between the continuous-flow group and the auto-DODS group was 1.8%, with a higher mean SpO_2_ in the continuous-flow group than in the auto-DODS group. The non-inferiority margin was set to 2.8%, which corresponded to 70% of the minimal clinically important difference (± 4%) in SpO_2_.^[4]^ Based on the SpO_2_ difference of 1.8% between continuous flow and the auto-DODS, the 1.4% standard deviation of the difference, and the noninferiority margin of 2.8%, we calculated, for an alpha level of 2.5% and a statistical power of 80%, that a sample size of 8 per group was needed. The formula used for the calculation is as follows: 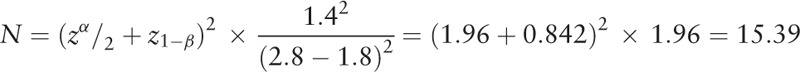


The sample size in this clinical study was set to 10 patients per group, in anticipation of a dropout rate of approximately 20%.

### 2.5 Processes and interventions

A steering committee including principal investigator and co-investigator are responsible for design, methodology and protocol amendments of the study. The principal investigator and co-investigators will conduct observations, inspections, and evaluations according to the following schedule.

After obtaining consent, the investigators will start the screening test. The patient characteristics to be assessed are summarized in Table 3. The principal investigator and co-investigators will identify patients who meet the inclusion criteria and do not meet the exclusion criteria and will include them as study subjects. On day 1, participants will be assigned to either an auto-DODS or continuous flow, which will be adjusted so that the level of SpO_2_ is the same in both conditions, and the participants will undergo polysomnography and complete a questionnaire (Table 4). On day 2, each participant will be assigned to the opposite device and will undergo polysomnography and a questionnaire once again. Participants will be checked for adverse events that are experienced during the treatment period until day 30.

**Table 3:**
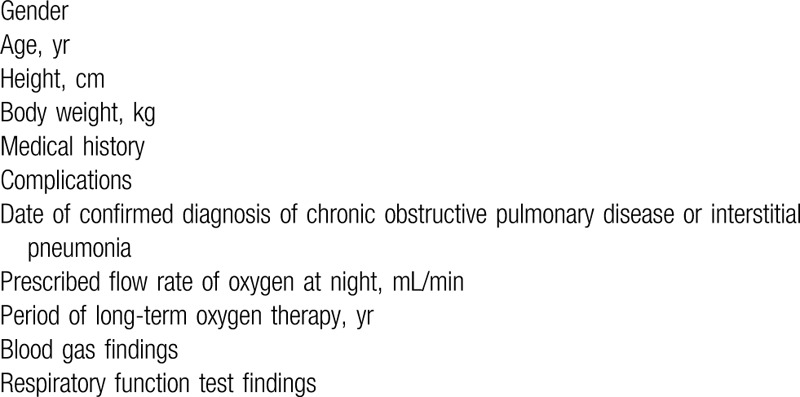
Patient characteristics.

**Table 4:**
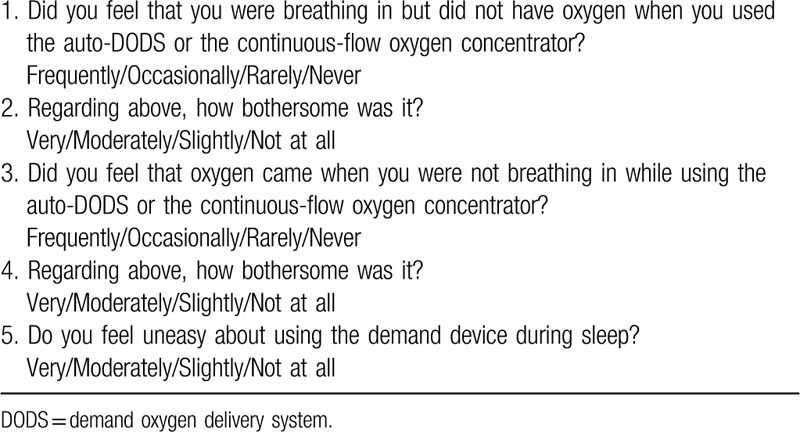
Questionnaire.

The principal investigator and co-investigators will create a “facility registration/user registration request form” and will send it to the data center. The principal investigator will receive and will retain the approval of the accredited clinical research review board and the administrator. After confirming that this trial has been published in the Japan Registry of Clinical Trials, the data center will perform facility registration and user registration based on the registration request forms and notifies the investigator of registration completion by E-mail.

The investigators will access the electronic data capture (EDC) using a user ID and password prepared in advance and input the case report data for each subject into the EDC. After confirming that the created case report form is correct, the principal investigator will electronically sign the EDC. The signed case report form will be printed and stored. If the data center inquires about input data, the principal investigator will confirm the content of the EDC and respond promptly.

Monitoring will be conducted regularly to confirm that this clinical trial is being conducted safely, in accordance with the study protocol version 2.0, and that data are being correctly collected. The principal investigator will appoint a monitoring supervisor and a monitor. The monitoring supervisor and the monitor must learn the regulatory requirements for clinical research and must fully understand the contents of the research protocol and monitoring procedures. During the study period, the monitor will directly check the consent sheets, medical records, and case reports. The monitor will perform these monitoring duties before, during, and after the trial.

To guarantee the quality of this clinical trial, we will evaluate whether this trial is being conducted in compliance with the study protocol and procedures, independent of the normal monitoring and quality control operations conducted by audit. The principal investigator will appoint an auditor and have that person carry out the audit in accordance with the audit procedure manual. The auditor will report the results of the audit to the principal investigator.

During monitoring, audits, and regulatory reviews related to clinical trials, the principal investigator, and medical institution will provide for direct review all records related to the clinical trial.

### 2.6 Comparison

Analysis will be performed after the use of the devices has ended in all cases and the data are fixed. In all efficacy evaluations, analysis of the full analysis set, comprising all subjects who use the test device, will be used as the main analysis, and analysis of the per-protocol set, including only subjects who followed the full protocol with no violations, will be performed as a reference. For the safety analysis, we will analyze a safety analysis set that includes all subjects who use a test device.

### 2.7 Statistical analysis

The primary aim of this trial is to test the noninferiority of the auto-DODS to a continuous-flow system in maintaining night-time SpO_2_. Based on the assumption that there is no carryover effect for treatment, we will analyze all eligible participants whose SpO_2_ data during the use of the 2 devices can be obtained. To test the noninferiority of the DODS, we will set the noninferior margin of the mean night-time value of SpO_2_ to Δ (= 2.8%). The null hypothesis H0 of the hypothesis test for noninferiority verification is *μ*1 ≦ *μ*_0_ − Δ, and the alternative hypothesis H1 is *μ*_1_ > *μ*_0_ − Δ, where *μ*_0_ is the population mean of SpO_2_ with a continuous-flow system, while *μ*_1_ is the population mean of SpO_2_ with an auto-DODS. If the lower limit of the estimated 95% CI for the difference of population means between the auto-DODS and continuous flow is above –Δ, we will regard the auto-DODS as noninferior. We will conduct a statistical test for noninferiority to calculate the *P*-value, with a 1-sided significance threshold of 2.5%.

We will also analyze secondary endpoints under the assumption that there is no carryover effect. The difference in the population means of SpO_2_ between the auto-DODS and continuous flow will be estimated, along with its 95% CI.

We will also conduct an analysis of variance that includes group, period, and device for the primary and secondary endpoints. Furthermore, as the subanalysis, we will estimate the difference in the population mean of each outcome, along with its 95% CI, between the auto-DODS and continuous flow using only the outcomes from the first period and will calculate the *P*-value based on Student *t* test or the Wilcoxon test.

## 3 Discussion

There has been only 1 report evaluating the nocturnal efficiency of a DODS.^[5]^ In that report, the DODS reduced oxygen usage by 60%. Although the DODS and continuous flow were not significant different in oxygenation or sleep quality, the continuous-flow system tended to be somewhat superior in oxygenation. Since the auto-DODS, in contrast to a conventional DODS, has 3 levels of sensitivity and a function that generates a pulsed flow in response to apnoea, we predict that the auto-DODS will not be inferior to continuous flow in oxygenation or sleep quality.

A limitation of this study is that we cannot assess the long-term prognostic impact of the auto-DODS. However, we believe that if the safety and efficacy of the auto-DODS are confirmed in 2 different disease states, COPD and interstitial pneumonia, it will be an advantage for many patients.

## Acknowledgment

The authors thank the staff of the Clinical and Translational Research Center at the Kobe University Hospital for their support in this study.

## Author contributions

**Conceptualization:** Kazuyuki Kobayashi, Takashi Omori, Takehiro Otoshi, Kanoko Umezawa, Naoko Katsurada, Masatsugu Yamamoto, Motoko Tachihara, Yoshihiro Nishimura.

**Data curation:** Tatsuya Nagano, Takehiro Otoshi, Kanoko Umezawa, Naoko Katsurada, Masatsugu Yamamoto.

**Formal analysis:** Tatsuya Nagano, Takashi Omori, Yoshihiro Nishimura.

**Funding acquisition:** Tatsuya Nagano, Yoshihiro Nishimura.

**Investigation:** Tatsuya Nagano, Takehiro Otoshi, Yoshihiro Nishimura.

**Methodology:** Tatsuya Nagano, Takashi Omori, Yoshihiro Nishimura.

**Project administration:** Tatsuya Nagano, Yoshihiro Nishimura.

**Resources:** Tatsuya Nagano.

**Software:** Tatsuya Nagano.

**Supervision:** Tatsuya Nagano, Kazuyuki Kobayashi, Takashi Omori, Yoshihiro Nishimura.

**Validation:** Tatsuya Nagano, Motoko Tachihara.

**Visualization:** Tatsuya Nagano.

**Writing – original draft:** Tatsuya Nagano, Kazuyuki Kobayashi, Takashi Omori.

**Writing – review and editing:** Tatsuya Nagano, Kazuyuki Kobayashi, Takashi Omori, Takehiro Otoshi, Kanoko Umezawa, Naoko Katsurada, Masatsugu Yamamoto, Motoko Tachihara, Yoshihiro Nishimura.
